# Women’s involvement in decision-making and association with reproductive health behaviors: findings from a cross-sectional survey in Niger

**DOI:** 10.1186/s12905-024-03115-x

**Published:** 2024-05-07

**Authors:** Sanyukta Mathur, Karen Kirk, Chaibou Dadi, Leanne Dougherty

**Affiliations:** 1 Population Council, 1015 15th St., NW, Washington, DC 20005 USA; 2 Conception Etudes Suivi Evaluation Appuis Formation, Niamey, Niger

**Keywords:** Empowerment, Decision-making, Social and behavior change programming, Reproductive health

## Abstract

**Background:**

Though women in Niger are largely responsible for the familial health and caretaking, prior research shows limited female autonomy in healthcare decisions. This study extends current understanding of women’s participation in decision-making and its influence on reproductive health behaviors.

**Methods:**

Cross-sectional survey with married women (15–49 years, *N* = 2,672) in Maradi and Zinder Niger assessed women’s participation in household decision-making in health and non-health issues. Analyses examined [1] if participation in household decision-making was associated with modern contraceptive use, antenatal care (ANC) attendance, and skilled birth attendance at last delivery and [2] what individual, interpersonal, and community-level factors were associated with women’s participation in decision-making.

**Results:**

Only 16% of the respondents were involved—either autonomously or jointly with their spouse—in all three types of household decisions: (1) large purchase, (2) visiting family/parents, and (3) decisions about own healthcare. Involvement in decision making was significantly associated with increased odds of current modern contraceptive use [aOR:1.36 (95% CI: 1.06–1.75)] and four or more ANC visits during their recent pregnancy [aOR:1.34 (95% CI: 1.00-1.79)], when adjusting for socio-demographic characteristics. There was no significant association between involvement in decision-making and skilled birth attendance at recent delivery. Odds of involvement in decision-making was significantly associated with increasing age and household wealth status, listening to radio, and involvement in decision-making about their own marriage.

**Conclusion:**

Women’s engagement in decision-making positively influences their reproductive health. Social and behavior change strategies to shift social norms and increase opportunities for women’s involvement in household decision making are needed. For example, radio programs can be used to inform specific target groups on how women’s decision-making can positively influence reproductive health while also providing specific actions to achieve change. Opportunities exist to enhance women’s voice either before women enter marital partnerships or after (for instance, using health and social programming).

## Background

While the world has made progress towards achieving the United Nations Sustainable Development Goals, there are still variations across regions. For example, in 2015–2021, an estimated 84% of births globally were assisted by skilled health professionals, increasing from 77% of births between 2008 and 2014 [1]. However, coverage in sub-Saharan Africa was 20% points lower than the global average. Key reproductive health (RH) indicators in Niger continue to lag behind other countries in the Sahel region of Africa. Nationally, high fertility rates (6.7 births per woman) [2] low contraceptive use (12.2% of married women currently using a modern method) and poor maternal health service use (32.8% of women attended four or more antenatal (ANC) visits and 29.3% of deliveries were conducted by skilled providers), contribute to persistently high RH-morbidity and mortality in the country. Further, national statistics belie considerable variation within the country as well; for instance, the fertility rates in Zinder and Maradi districts are much higher than the national average at 8.5 and 8.4 births per women, respectively [3]. Evidence suggests that women’s empowerment, and specifically, their engagement in household decision-making, can positively influence a range of RH outcomes [4–6]. However, women in Niger are often not involved in health care decisions in the household and men are often the primary or final decision-makers, particularly when those decisions have financial implications [3, 7].

Previous investigations of women’s decision-making as a predictor for a range of RH outcomes in diverse African contexts have found mixed effects. Research on modern family planning (FP) use found that women with greater wealth, educated partners, and decision-making power were more likely to have used contraceptives [4] and autonomous decision-making among women for their own health increased the odds of modern FP compared to women who were not involved in such decisions [8]. For instance, a study of 32 sub-Saharan African countries found that female adolescents who exhibit “reproductive health decision-making capacity”—as measured by self-reported ability to refuse sexual intercourse and ask partner to use a condom—had higher odds of using contraceptives and this increased with age, wealth, education, urban dwelling and if the adolescent was cohabitating and not married [9]. Additional research on women’s decision-making found that ANC attendance [10] and facility-based delivery [10, 11] were more likely behaviors among women with decision-making power. Recently published studies examining how decision-making ability among married women influences RH outcomes like contraceptive use [4] and skilled birth attendance (SBA) [6] are reliant on pooled national-level data, which includes data from Niger from 2012.

Further, the literature investigating factors associated with/enabling women’s participation in household decision-making among married women also notes varied results. Previous research has examined determinants of women’s decision-making using Demographic Health Surveys and found that older age at marriage among adolescents in Niger [12], and age, education, religion, wealth, geographical region, urban residence and education in Nigeria [13] are associated with greater decision-making among married women. A regional study using pooled national-level data from 27 sub-Saharan African countries found that rural living, no education, practicing Islam, not working, and uneducated partners negatively influence a women’s involvement in RH decisions [14]. Whereas a study from Burkina Faso found that while free RH and FP services and commodities improved access to health services by facilitating the negotiation processes between women and their families (i.e., the husband’s brother or parents), prevalent social norms and gender inequalities continued to limit women’s decision-making power [15]. Several studies within the FP sphere have found that couples’ communication—a component of household decision-making—is linked to women’s involvement in decision-making, and that promotion of intra-couple communication as a high impact practice that can lead to better RH outcomes for women [16]. It is unclear what factors influence married women’s participation in household decision-making in Niger and if these could serve as potential points of intervention to enhance women’s empowerment and improve RH outcomes.

**Fig. 1:**
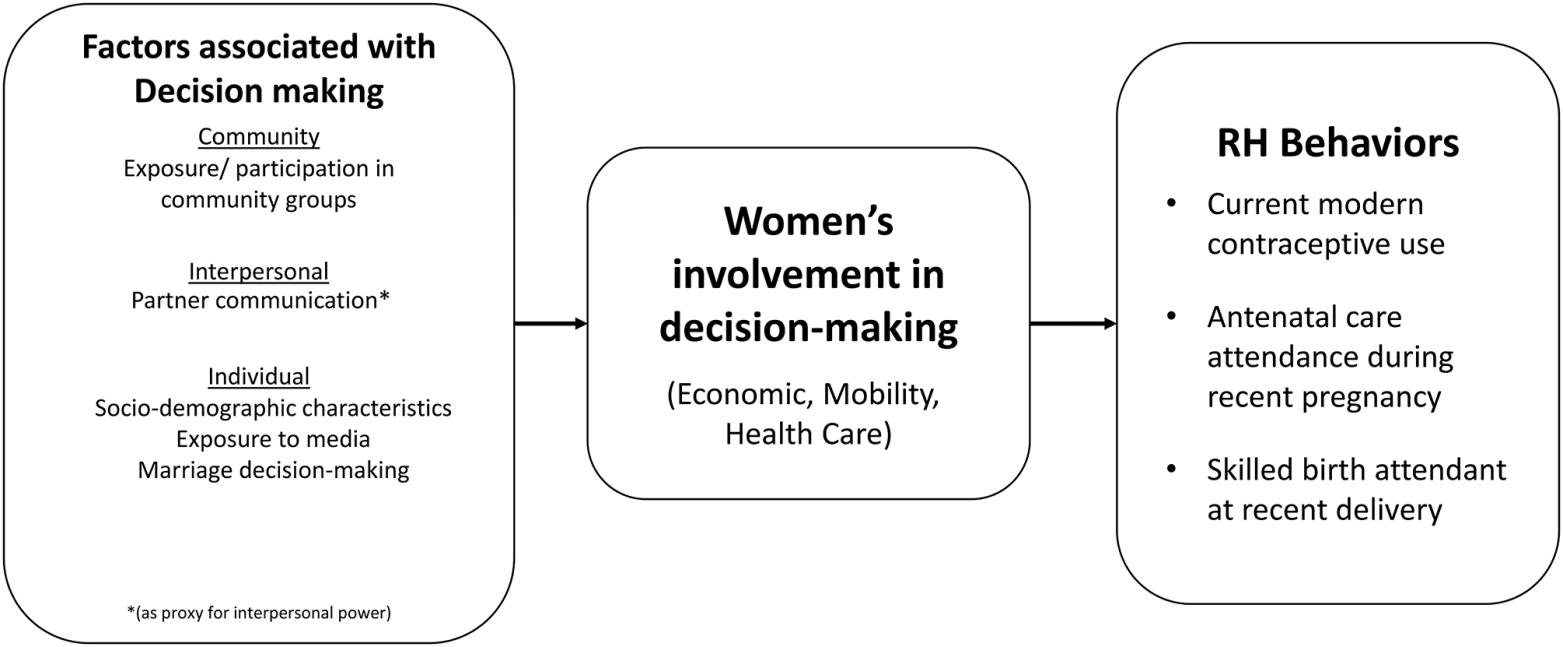
Conceptual Framework on Women’s Involvement in Decision-making and RH Behaviors (Informed by the reproductive empowerment [17] and socio-ecological frameworks [18])

The present study aims to extend current understanding of married women’s participation in decision making and its influence on RH behaviors in Niger. Our analysis was guided by the conceptual framework (Fig. 1) which draws upon existing theoretical and conceptual frameworks [17, 18] and illustrates factors hypothesized to be associated with women’s involvement in decision making and RH behaviors. Informed by prior literature, we conceptualized women’s involvement in decision-making, a key aspect of women’s empowerment [19], by their capability to contribute to household decision-making on multiple dimensions. As empowerment in one dimension does not necessarily indicate empowerment in another, we use measures that capture decision-making regarding economic, mobility, and personal healthcare [20]. Further, as empowerment is contextually driven, we use an expanded definition of decision-making to include either autonomous decision-making or joint decision-making. A recent qualitative study confirmed that women in Niger rarely make health decisions autonomously though they were often involved in the process of decision-making related to health care seeking [7]. Prior research has noted the lack of clarity or contextual appropriateness of dichotomies like autonomous vs. joint-decision-making. Women who make decisions autonomously may have no other option or support and women who participate in joint decision-making may be playing an active/cooperative role in the decision [21]. With this framing of women’s engagement in decision-making, we explore associations with select RH behaviors (modern contraceptive use, ANC attendance and facility-based delivery) and then examine correlates of women’s engagement in decision-making. To explore the characteristics of women who engage in decision-making, we examined individual, interpersonal, and community-level factors using the reproductive empowerment [17] and socio-ecological frameworks [18]. With this quantitative exploration, we hope to contribute to the growing literature in the Sahel region on women’s empowerment and decision-making and its associations with RH behaviors using recently collected sub-national data, as well as identify opportunities to inform empowerment-focused programming in the region.

## Methods

### Study design and sites

We use data from a baseline survey conducted as part of an evaluation of the U.S. Agency for International Development (USAID) supported Resilience in the Sahel Enhanced (RISE) II program by Breakthrough RESEARCH, USAID’s flagship global SBC evidence generation project. This cross-sectional household survey, conducted in March 2021, interviewed married women of reproductive age (15–49 years) in Maradi and Zinder regions of Niger. Located in western Africa, Niger is a landlocked country and a vast majority of it includes the Saharan desert terrain. The Maradi and Zinder regions are located along the southern border, where ongoing conflicts in the region have led to tens of thousands of refugees crossing into Maradi, Niger from Nigeria over the last few years [22]. The economy in the study area is largely based on subsistence agriculture. Under the RISE II activity in the Maradi and Zinder regions, community-based groups promote health behaviors and address development challenges. Specifically, these groups address male engagement and couples’ communication, and support income generating activities and savings and loan groups, which support the social and economic empowerment of women.

### Sampling

**Fig. 2:**
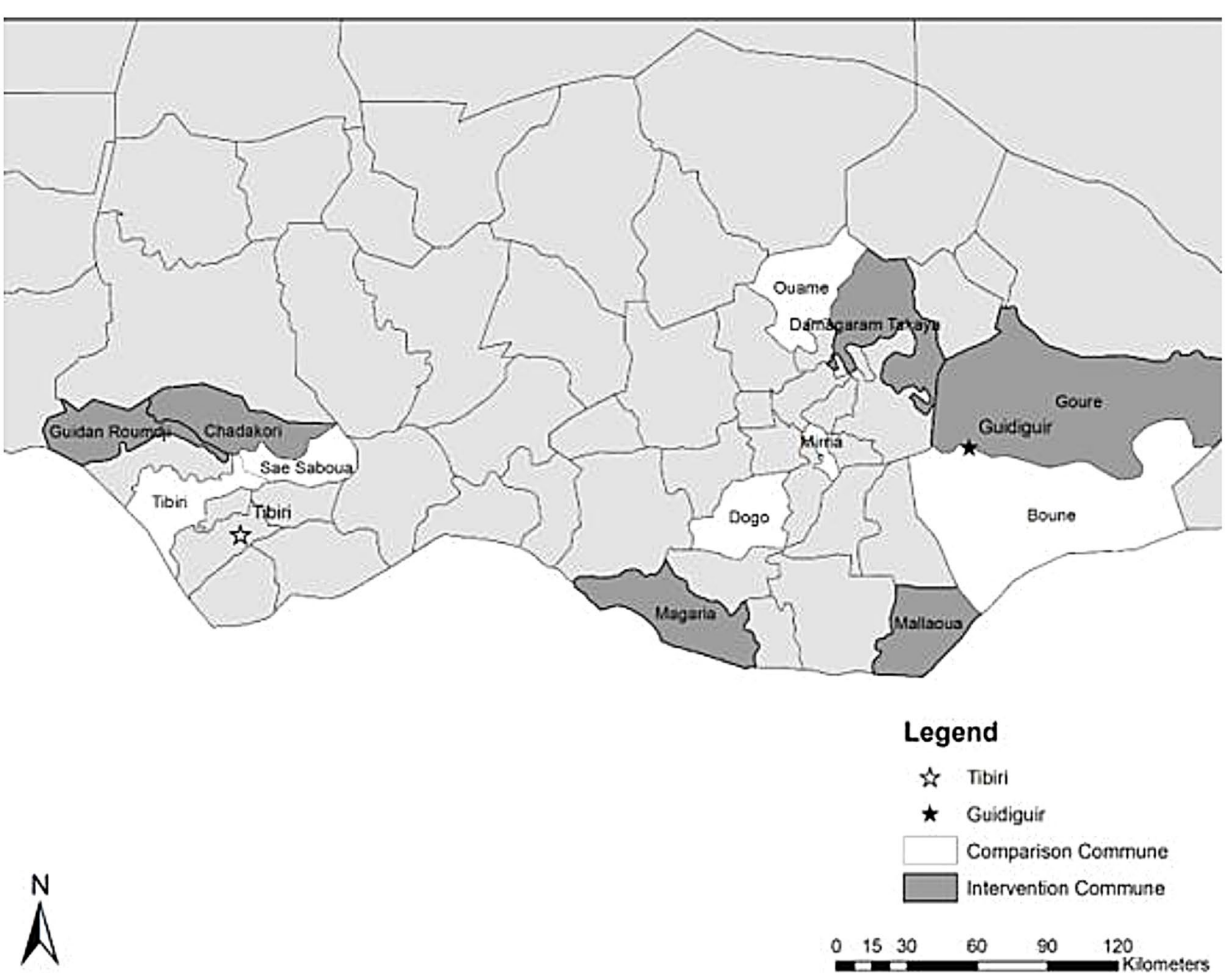
Map of RISE II study areas in Niger

The sampling procedure for this survey involved three stages: 1) six of the 18 intervention communes (four in Zinder and two in Maradi regions) were randomly selected for this survey and six control communes with similar characteristics were selected as shown in Fig. 2) all enumeration areas identified in the 2012 General Census were listed for selected communes; 3) probability proportion to size was used to select enumeration areas for each commune starting at a random point and systematically selecting areas using a fixed sampling interval and randomly selected one eligible woman per household. Our analysis relied on the data collected from married women aged 15–49 who completed the quantitative survey which was administered via digital tablets using SurveyCTO.

### Measures

Table 1 presents measures for variables used in our analyses of factors related to women’s involvement in three areas of decision-making and how it relates to desired RH behaviors. Individual level variables included socio-demographic characteristics (e.g., age, any education, marital status, number of children under five years, and wealth (terciles)), having listened to the radio in the last week and women’s involvement in the decision for her to marry. The interpersonal level variable assessed partner communication on RH issues, and community level variables included women’s involvement in community groups. Our outcomes of interest were three RH behaviors: modern contraceptive use, attending four or more ANC consultations, and skilled birth attendance. The rationale for not applying the World Health Organization ANC model of at least eight or more ANC visits was because fewer than 1% of all women interviewed who gave birth in the last five years reported attending eight or more ANC visits.

**Table 1:** Description of variables

Variable	Type of Measure	Description
Age	Continuous	Single continuous item
Education	Dichotomous	Single item, defined as no education or any education, based on the question “Have you gone to school?”
Marital status	Categorical	Defined as married/monogamous, or married/polygamous, based on the questions “Are you currently married, living with a man like you are married?” and “Does your (husband/partner) have other wives?”
Number of children under 5 years	Categorical	Single item. Defined using the reported number of children under 5 years of age were made into categories for: 1 child, 2 children, and 3 or more children
Wealth status	Categorical	Categorical, household wealth terciles constructed using Equity tool methods [23] for Niger.
Listened to the radio	Dichotomous	Single item defined combining responses of “at least once a week” and “less than once a week” to show the respondent “listens to the radio” and “not at all” and “refused” to reflect the respondent “does not listen to radio”
Women’s involvement in community group	Dichotomous	Single item used to create a dichotomous variable about involvement in any community group. Respondents who reported being involved in any one or more community groups are coded as “involved,” the rest were coded as “not involved.”
Woman’s involvement in marriage decision	Dichotomous	Single item used to create dichotomous variable for woman’s involvement in the decision for her to marry. Respondents who reported being included in the decision that she marry are defined as “involved,” the rest were coded as “not involved.”
Perceived ability to communicate with partner on RH issues	Dichotomous	Two items about women’s perceptions of how easy it is to engage their husbands in conversations about ANC attendance and facility delivery were used to create a dichotomous variable. Those who answered, “not difficult at all” for either item were coded as perceiving these conversations as “not difficult,” the rest were coded as “Difficult.”
Woman’s involvement in decision-making	Dichotomous	Variable created using three items on a woman’s involvement in decision-making about: (1) making a large purchase, (2) visiting her family/parents, and (3) decisions about her own healthcare. Respondent was categorized as “involved” in decision-making if she answered either that she herself made the decision or that she and her partner made the decision jointly for all three decision types, the rest were coded as “not involved.”
Modern contraceptive use	Dichotomous	Responses to “Are you currently doing something or using a method to delay or avoid pregnancy?/What method are using??” were separated and used to create a new binary variable on Modern contraceptive use in which “Female Sterilization,” “male sterilization,” “IUD,” “injectable,” “implant,” “pill,” “male condom,” and “female condom” responses are considered “using a modern method”, the rest (“LAM,” or lactation amenorrhea “Rhythm,” “withdrawal,” and “other”) were coded as “not using a modern method.”
ANC attendance during recent pregnancy	Dichotomous	Using binary Yes/No answers about consulting someone for ANC during recent pregnancy in the last five years and a continuous variable of “how many visits” we created a binary variable for attending < 4 and 4 + ANC visits during recent pregnancy
Skilled Birth Attendance (SBA) during recent delivery	Dichotomous	Respondents provided information on anyone who helped them with the delivery of their children in the last five years. Multiple responses were separated, and “other” responses were used to create a new categorical variable to reflect the highest qualified attendant present at delivery. Next, this was dichotomized into a new variable for SBA during recent delivery (Yes/No).

### Analysis

Statistical analysis was conducted using Stata v16 [24]. First, we ran correlation matrices and found a moderate level of collinearity across the three decision-making items and determined that it would not be appropriate to include all three items independently in the analysis. We therefore constructed a composite decision-making variable as described in Table 1. We then confirmed variable independence with the remaining predictor variables. We ran unadjusted logistic regressions to test associations between the decision-making composite measure and RH dependent variables. We then ran multivariate logistic regression models to assess if women’s involvement in decision-making was associated with the odds of modern contraceptive use, ANC attendance, and SBA, adjusted for socio-demographic variables as well as factors significantly associated with women’s involvement in decision-making. Regression models with each individual decision-making item were run separately (data not shown) and results were consistent with the composite decision-making variable model. Next, to inform empowerment-focused programming in the region, we conducted a descriptive analysis to assess variability of factors associated with decision-making. We tested for associations between factors associated with decision-making and women’s involvement in decision making. We used chi-squared tests for dichotomous decision-making antecedent variables and the Mann-Whitney test for continuous variables. We then constructed a multivariate logistic regression model with our composite variable on women’s involvement in decision making as the dependent variable. The standard errors were adjusted by strata and commune to account for the sampling approach.

### Ethics

The Ministry of Public Health National Ethics Committee for Health Research in Niger provided approval for the study and consent forms (No. 017/2020/CNERS). The study also received approval from the Population Council Institutional Review Board in the United States (No. 934). The relevant ethical approval and consent details were received and are available on request by the editor or editorial office. The informed consent was read to the participants by a research assistant trained in ethical human subjects research. The consent form used lay language and the participants were given the opportunity to ask questions about the study before giving consent. Study participants provided informed consent by marking agreement (in the form of an X) without using their signature. This procedure is prevalent in similar study settings and allows for the inclusion of illiterate individuals or those without defined signatures. Women interviewed between the ages of 15 and 17 years were emancipated minors and therefore parental and/or legal guardian consent was not required. In addition, all methods were carried out in accordance with relevant guidelines and regulations and with the 1964 Helsinki declaration and its later amendments or comparable ethical standards.

## Results

A total of 2,672 women participated in the survey. These women were on average, 31 years old, largely uneducated (85.4%), and mothers of one or more children (93.8%). In our sample, most women were not exposed to radio (65.2%) or community groups (69.1%) (Table 2). Study participants generally tended to not be involved in key household decisions, only 1–2% made any of the three household decisions described in Table 2 on their own and 20–30% reported joint decision making on their own health, large purchases, or family visits (results not shown). In our composite measure of women’s involvement in decision-making, only 16% were involved in either joint or autonomous decision-making on all three types of decisions in the household (Table 2). Current modern contraceptive use was reported by only 23% of women, while 54% reported 4 or more ANC visits for their most recent pregnancy, and 59% reported SBA for their most recent delivery.

**Table 2:** Summary of participant characteristics

	Women (*N* = 2,672)	
	*n*	%
Age in years (mean, SD, range)	31.0 *±* 6.7 (15–49)	
Any education		
No	2,283	85.4%
Yes	389	14.6%
Marital status		
Married, monogamous	1,693	63.4%
Married, polygamous	979	36.6%
Number of children under 5 years		
0	165	6.2%
1	747	28.0%
2	1,488	55.7%
3+	272	10.2%
Wealth status		
Lowest tercile	903	33.8%
Middle tercile	884	33.1%
Richest tercile	885	33.1%
Listened to the radio		
No	1,742	65.2%
Yes	930	34.8%
Women’s involvement in community group		
No	1,655	61.9%
Yes	1,017	38.1%
Woman’s involvement in marriage decision		
No	1,683	63.0%
Yes	989	37.0%
Woman’s involvement in decision-making		
No	2,249	84.2%
Yes	423	15.8%
Modern contraceptive use		
No	2,048	76.7%
Yes	624	23.4%
ANC attendance (4 + visits)	(*N* = 2,506)	
No	1,161	46.3%
Yes	1,345	53.7%
SBA	(*N* = 2,507)	
No	1,017	40.6%
Yes	1,490	59.4%

### Involvement in decision-making and influence on RH outcomes

Table 3 presents analysis on the association of women’s involvement in decision-making and three RH outcomes. Both unadjusted and adjusted regressions indicated that a woman’s involvement in key household decisions was associated with increased odds of current modern contraceptive use [OR: 1.64 (95% CI: 1.26–2.14); aOR: 1.36 (95% CI: 1.06–1.75)]. Respondents’ involvement in decision making was also associated with increased odds of four or more ANC visits during her recent pregnancy [aOR: 1.34 (95% CI: 1.01–1.79)] compared to those who were not involved in household decisions about making a large purchase, visiting her family/parents, and decisions about her own healthcare. Involvement in decision-making was not significantly associated with having an SBA during a woman’s most recent delivery.

**Table 3:** Results from logistic regression models of women in decision-making and RH outcomes

	*N*	OR (95% CI)	*p*-value	aOR^^^ (95% CI)	*p*-value
Modern contraception use	2,672	1.64 (1.26–2.14)	0.000	1.36 (1.06–1.74)	0.016
4 + ANC visits during recent pregnancy	2,506	1.30 (0.98–1.72)	0.073	1.34 (1.01–1.79)	0.047
SBA during recent delivery	2,507	1.09 (0.84-1.42)	0.505	0.99 (0.77-1.29)	0. 982

^^^Adjusted for age, any formal education, number of children under 5, marital status. wealth status, radio listenership, involvement in marriage decision-making

### Characteristics of women involved in decision-making

To inform empowerment-focused programming in the region, we examined characteristics of women involved in decision-making. As only a minority of women in our study population were involved in key household decisions as defined in this analysis, we examined the profiles of the women who were involved in such decision-making as compared to those who were not involved. Table 4 presents bivariate and multivariate associations between involvement in household decision-making and a range of individual, interpersonal, and community characteristics that may influence women’s empowerment. Bivariate results show that age, marital status, wealth status, radio listenership, and involvement in decision-making regarding their own marriage were associated with involvement in household decision-making. Findings from the logistic regression model examining these associations (Table 4) show that being in a polygamous marriage [aOR: 1.41 (95% CI: 1.09–1.83)], highest household wealth tercile: aOR 2.3 (95% CI: 1.49–3.56), having listened to the radio [aOR: 1.55 (95% CI: 1.17–2.06)] and involvement in own marriage decision [aOR: 2.62 (95% CI: 2.07–3.33)] were associated with women’s involvement in decision-making, while age, education, number of children, involvement in community groups and woman’s perceived ability to communicate with partner on RH issues were not statistically associated with women’s involvement in decision-making.

**Table 4:** Correlates of involvement in decision-making

	Involved in decision-making (Yes)	*p*-value	aOR (95% CI)	*p*-value
	*N* (%)			
Age in years (mean, SD, range)	32.15 *±* 6.6 (15–49)	0.000	1.01 (1.0-1.02)	0.128
Any education		0.616		
No	357 (84.4%)			
Yes	66 (15.6%)			
Marital status		0.000		
Married, monogamous	226 (53.4%)		reference	
Married, polygamous	197 (46.6%)		1.41 (1.09–1.83)	0.011
Number of children under 5 years		0.368		
0	18 (4.3%)			
1	115 (2727%)			
2	242 (57.2%)			
3+	48 (11.4%)			
Wealth status		0.000		
Lowest tercile	90 (21.3%)		reference	
Middle tercile	126 (29.8%)		1.42 (0.97–2.10)	0.071
Richest tercile	207 (48.9%)		2.30 (1.49–3.56)	0.000
Listened to the radio		0.000		
No	224 (53.0%)		reference	
Yes	199 (47.0%)		1.55 (1.17–2.06)	0.003
Women’s involvement in community group		0.170		
No	245 (57.9%)			
Yes	178 (42.1%)			
Woman’s involvement in marriage decision		0.000		
No	186 (44.0%)		reference	
Yes	237 (56.0%)		2.62 (2.07–3.33)	0.000
Perceived ability to communicate with partner on RH issues		0.105		
Not difficult	395 (93.4%)	0.02	1.23 (0.80–1.90)	0.349
Difficult	28 (6.6%)		Reference	

## Discussion

Understanding determinants and associated outcomes of women’s involvement in decision-making in Niger can provide helpful insights and important cultural and practical context to improve the social position and reproductive well-being of married women. In this paper, guided by the reproductive empowerment [17] and socio-ecological frameworks [18], we explore both how decision-making is linked to RH behaviors and what factors are associated with decision-making in a sub-national context in Niger to provide insights for social and behavior change programs in the region.

In this study, we found that women’s involvement in decision-making is associated with improved RH outcomes for contraceptive use and ANC. Our findings are supported by other research in the region that shows similar positive associations between women’s empowerment/decision-making and reproductive health outcomes [4, 5, 9, 14]. Our study contradicts a previous analysis of pooled DHS data—including data from Niger—that showed that SBA was higher among women who are involved in decision-making compared to those who were not [6]. However, additional analyses from Niger suggests that distance to health facility can also influence SBA [6, 25]. It is possible that the magnitude of the association could be moderated by distance, if women in our sample had to travel long distances to reach a health facility, as is often the case in Niger [26]. We also found that women who are involved in decision-making tend to be wealthier, or in a polygamous union. These expected demographic factors were significantly associated with women’s involvement in decision-making and are seen in similar studies from across the sub-Saharan region [4, 8, 9, 12, 27]. In addition, women in our sample who were involved in household decision-making had listened to radio programming or had participated in their own marriage decision, compared to women who were not involved in decision-making. These last two insights might offer potential avenues for intervention—the former, which could be implemented to reach married and unmarried women, and the latter requiring action before marriage through family and community intervention [28].

While this study found a positive association with having listened to the radio and involvement in decision-making, this finding is not in alignment with prior analysis that found no observed association between decision-making capacity and having listened to the radio [29]. This difference could be due to the lower penetration of mass media in our study setting; only 35% of our sample was exposed to radio. In addition, TV exposure was so low in the sample, that we did not include it in our analysis or summary findings [30]. Prior research shows that women in the Maradi and Zinder regions of Niger have primarily relied on the use of interpersonal communication with health providers as their source of information on RH [30]. While the role of passive health communication, through TV or radio has diminished in recent years, recent research in the country has found that targeted radio programming can be an empowering resource for women and may be appropriate for hard-to-reach populations [31].

We also find that women who reported being involved in their own marriage decisions were also involved in current household decision-making. Engaging with families and communities to address drivers of child marriage [32] could lay the groundwork for women’s better involvement in decision-making and subsequently improve RH behaviors. Previous qualitative research from the Zinder region of Niger found that young newly married women are unable to delay pregnancy and pursue opportunities such as education and economic pursuits due to structural factors related to lack of mobility and autonomy [33]. Our study findings suggest that women who report being involved in the decision to get married are also involved in household decisions later, though programs should carefully consider local context and meaning of ‘participation in marriage decision’. Previous qualitative findings indicate that in the Nigerien context marriage is seen as ‘inevitable’, and programs aimed at increasing involvement in one’s own marriage decision should focus on when to marry rather than whether to marry, as even for women who see themselves as having played a role in her marriage decision find the final decision lies with her parents [34].

Through our study approach, a strength is that we highlight the need to consider how decision-making is measured in Niger. Most research emphasizes autonomous decision-making as a marker for women’s empowerment [35–37]. However, within this context where so few women report making any household decisions, autonomous decision-making may not be a contextually relevant measure of empowerment (yet). Only 16% of our sample were involved in *any* decision-making. Prior qualitative research in the study communities showed that women do participate in decision-making within the household through distinct pathways and varying levels of engagement with their spouses [7]. In a context like Niger, strategies may be needed to provide both husbands and wives with information and skills to promote communication and collaboration regarding decision-making. Program efforts like the RISE II initiative are attempting to address women’s empowerment directly to improve the range of health and development outcomes for women and their families, as well as engaging men and addressing gender norms [38]. These efforts are well-warranted and there are some indications that efforts to increase women’s access to resources improves women’s empowerment and decision-making power in the region [39]. The majority of women in our sample perceived no difficulty in being able to communicate with their partner/spouse on RH issues. Couples’ communication is a key aspect of women’s engagement in household decision-making and in decisions and behaviors related to RH outcomes and has been recognized and well documented for decades within the FP field [16, 40–42]. For instance, a study among young couples in Niger found that those who discussed contraceptive use were more likely to report using contraception [43]. Furthermore, another study in Niger found married men who believe that they alone should make decisions about FP use had 39% lower odds of reporting FP use [44]. Similar findings were observed related to ANC in the Zinder region of Niger, where women who reportedly received husbands’ advice about attending ANC were more likely to attend ANC and adhere to iron-folic acid supplementation than women who did not [45]. However, it is important to note that receiving advice from a husband does not necessarily indicate that this interaction implied joint decision making. Although we were unable to fully explore these dynamics in our study, for married women in Niger, couple interventions may be a promising strategy to increase discourse on health and engagement in health-related decision-making within the household. However, more research on these promising approaches, particularly, those that engage men and women to modify gender norms around communication related to and decision making between spouses is needed [46].

### Limitations

The main limitation of this study is that it relies on self-reported cross-sectional data. By analyzing data from one point in time, we are unable to make any inferences or causal connections from our findings. Additionally, questions related to decision making did not include a temporal reference. Therefore, we assumed the response at the time of the survey was the same as the time the respondent practiced the health behavior. While household decision-making inherently involves participation of other household members, most commonly the women’s husbands, this study does not look at men’s views on women’s involvement in decision-making nor on their desired RH behaviors; the nuance between joint-decision-making and perception of joint decision-making may need to be further investigated. Future analyses may want to tease out associations between decision-making and outcomes when looking at autonomous decision-making vs. joint decision-making, though as we’ve argued, autonomous decision-making might not be empowering for women in this context where less than 2% of women in our sample solely made key household decisions. In addition, our study only measured the association of perceived ability to communicate with partner on ANC and SBA outcomes. Future studies should also consider the perceived ability to communicate with partners on FP outcomes. Finally, as seen elsewhere, partner communication is an important aspect of women’s involvement in decision-making that our data did not measure and therefore our analysis could not explore; future work around decision-making should measure and explore the role of couple’s communication on women’s involvement in household decisions and how interventions aimed at strengthening couple’s communication is associated with decision making and health outcomes [7].

## Conclusions

Our study found that despite overall low engagement in decision making, women who were involved in decision making had increased odds of using FP and ANC services and that individual, and interpersonal level factors influenced a woman’s involvement in decision making. These findings suggest that programs should actively engage actors at various levels of the socioecological model to strengthen women’s decision making and use of RH services. Younger and poor women warrant particular attention programmatically given their likely lack of agency and resources that would enable them to participate in household decision making. Enhancing young women’s participation in marriage choice may be an avenue for change. Additionally, we found a strong association between women’s exposure to radio programs and decision making. Social and behavior change programs should consider identifying opportunities to increase women’s exposure to health and social programming through radio programs. In a context with low literacy, low media penetration, and high gender inequality, it is possible that providing accessible information, supported by strategies that encourage couple communication and decision-making, could engender desired reproductive health outcomes.

## Data Availability

The datasets used and/or analyzed during the current study available from the corresponding author on reasonable request.

## Abbreviations

ANC: Antenatal care
FP: Family planning
RH: Reproductive health
RISE: Resilience in the Sahel Enhanced
SBA: Skilled birth attendance
USAID: U.S. Agency for International Development

## References

[1] United Nations. The Sustainable Development Goals Report [Internet]. New York, N.Y., USA. 2022. https://unstats.un.org/sdgs/report/2022/The-Sustainable-Development-Goals-Report-2022.pdf.

[2] World Bank. World Bank Development Indicators. [cited 2022 Aug 29]. Fertility rate, total (births per woman). https://data.worldbank.org/indicator/SP.DYN.TFRT.IN.

[3] Institut National de la Statistique (INS) (2013). Enquête Démographique Et De Santé Et à Indicateurs multiples Du Niger 2012.

[4] Yaya S, Uthman OA, Ekholuenetale M, Bishwajit G (2018). Women empowerment as an enabling factor of contraceptive use in sub-saharan Africa: a multilevel analysis of cross-sectional surveys of 32 countries. Reprod Health.

[5] Prata N, Fraser A, Huchko MJ, Gipson JD, Withers M, Lewis S (2017). Women’s empowerment and Family Planning: a review of the literature. J Biosoc Sci.

[6] Dickson KS, Adde KS, Ameyaw EK. Women empowerment and skilled birth attendance in sub-Saharan Africa: A multi-country analysis. Kabir E, editor. PLoS ONE. 2021;16(7):e0254281. 10.1371/journal.pone.0254281 PMC8263257 34234362

[7] Chace Dwyer S, Mathur S, Kirk K, Dadi C, Dougherty L (2022). When you live in good health with your husband, then your children are in good health … a qualitative exploration of how households make healthcare decisions in Maradi and Zinder Regions, Niger. BMC Public Health.

[8] Butler MG, Walker M, Pablo LA, Bartels SA (2021). Relationship between women’s decision-making power over their own health care and use of modern contraception in the Democratic Republic of the Congo: a cross-sectional secondary data analysis. BMC Women’s Health.

[9] Ahinkorah BO, Hagan JE, Seidu AA, Sambah F, Adoboi F, Schack T (2020). Female adolescents’ reproductive health decision-making capacity and contraceptive use in sub-saharan Africa: what does the future hold? Oladimeji O. Editor PLoS ONE.

[10] Sripad P, Warren CE, Hindin MJ, Karra M (2019). Assessing the role of women’s autonomy and acceptability of intimate-partner violence in maternal health-care utilization in 63 low- and middle-income countries. Int J Epidemiol.

[11] Tekelab T, Yadecha B, Melka AS (2015). Antenatal care and women’s decision making power as determinants of institutional delivery in rural area of Western Ethiopia. BMC Res Notes.

[12] Tomar S, Johns N, Challa S, Brooks MI, Aliou S, Abdoul-Moumouni N (2021). Associations of Age at Marriage with Marital decision-making Agency among adolescent wives in rural Niger. J Adolesc Health.

[13] Osamor P, Grady C (2018). Factors Associated with women’s Health Care decision-making autonomy: empirical evidence from Nigeria. J Biosoc Sci.

[14] Darteh EKM, Dickson KS, Doku DT. Women’s reproductive health decision-making: A multi-country analysis of demographic and health surveys in sub-Saharan Africa. Withers MH, editor. PLoS ONE. 2019;14(1):e0209985. 10.1371/journal.pone.0209985 PMC6326492 30625212

[15] Beaujoin C, Bila A, Bicaba F, Plouffe V, Bicaba A, Druetz T (2021). Women’s decision-making power in a context of free reproductive healthcare and family planning in rural Burkina Faso. BMC Women’s Health.

[16] High Impact Practices. Promoting healthy couples’ communication to improve reproductive health outcomes [Internet]. 2022 [cited 2022 Nov 16]. https://www.fphighimpactpractices.org/briefs/couple-communication/.

[17] Edmeades J, Mejia C, Parsons J, Sebany M. A Conceptual Framework for Reproductive Empowerment: Empowering Individuals and Couples to Improve their Health (Background Paper) [Internet]. Washington, DC: International Center for Research on Women; 2018. https://www.icrw.org/wp-content/uploads/2018/10/Reproductive-Empowerment-Background-Paper_100318-FINAL.pdf.

[18] Bronfenbrenner U (1977). Toward an experimental ecology of human development. Am Psychol.

[19] Kabeer N, Resources (1999). Agency, achievements: reflections on the measurement of women’s empowerment. Dev Change.

[20] Pratley P (2016). Associations between quantitative measures of women’s empowerment and access to care and health status for mothers and their children: a systematic review of evidence from the developing world. Soc Sci Med.

[21] Hinson L, Edmeades J, Murithi L, Puri M (2019). Developing and testing measures of reproductive decision-making agency in Nepal. SSM - Popul Health.

[22] UNHCR Niger - Operational Update [Internet]. UNHCR. 2022. https://data.unhcr.org/en/documents/details/94641.

[23] Niger Equity Tool [Internet]. EquityTool. 2016. Available from: equitytool.org.

[24] StataCorp (2019). Stata Statistical Software: release 16. College Station.

[25] Ameyaw EK, Dickson KS (2020). Skilled birth attendance in Sierra Leone, Niger, and Mali: analysis of demographic and health surveys. BMC Public Health.

[26] Blanford JI, Kumar S, Luo W, MacEachren AM (2012). It’s a long, long walk: accessibility to hospitals, maternity and integrated health centers in Niger. Int J Health Geogr.

[27] Castro Lopes S, Constant D, Fraga S, Bique Osman N, Correia D, Harries J. Socio-economic, demographic, and behavioural determinants of women’s empowerment in Mozambique. Darteh EKM, editor. PLoS ONE. 2021;16(5):e0252294. 10.1371/journal.pone.0252294 PMC8162630 34048468

[28] Silva M, Kassegne S, Nagbe RHY, Babogou L, Ezouatchi R, Ado AL et al. Changing the script: Intergenerational Communication about sexual and Reproductive Health in Niamey, Niger. J Health Communication. 2022;1–9. 10.1080/10810730.2022.2160527 36567672

[29] Seidu AA, Ahinkorah BO, Hagan JE, Ameyaw EK, Abodey E, Odoi A (2020). Mass Media exposure and women’s Household decision-making capacity in 30 sub-saharan African countries: analysis of demographic and health surveys. Front Psychol.

[30] Dougherty L, Turk L, Jani N, Dadi C. Evaluation of RISE II integrated social and behavior change activities in Niger: Baseline report. Ensure Sexual and Reproductive Health, Rights, and Choices [Internet]. 2022; https://knowledgecommons.popcouncil.org/focus_sexual-health-repro-choice/76.

[31] Heywood E (2020). Radio Journalism and women’s empowerment in Niger. Journalism Stud.

[32] Psaki SR, Melnikas AJ, Haque E, Saul G, Misunas C, Patel SK (2021). What are the drivers of child marriage? A conceptual Framework to Guide policies and programs. J Adolesc Health.

[33] Samandari G, Grant C, Brent L, Gullo S (2019). It is a thing that depends on God: barriers to delaying first birth and pursuing alternative futures among newly married adolescent girls in Niger. Reprod Health.

[34] Saul G, Diarra A, Melnikas AJ, Amin S (2020). Voice without Choice? Investigating adolescent girls’ Agency in Marital decision-making in Niger. Progress Dev Stud.

[35] Kebede AA, Cherkos EA, Taye EB, Eriku GA, Taye BT, Chanie WF. Married women’s decision-making autonomy in the household and maternal and neonatal healthcare utilization and associated factors in Debretabor, northwest Ethiopia. Spradley FT, editor. PLoS ONE. 2021;16(9):e0255021. 10.1371/journal.pone.0255021 PMC8476028 34570781

[36] Osamor P, Grady C. Women’s autonomy in health care decision-making in developing countries: a synthesis of the literature. IJWH. 2016;191. 10.2147/IJWH.S105483 PMC4908934 27354830

[37] Dyson T, Moore M (1983). On Kinship structure, female autonomy, and demographic behavior in India. Popul Dev Rev.

[38] USAID. USAID Resilience in the Sahel Enhanced (RISE) II Technical Approach Working Paper [Internet]. Washington, DC: USAID. 2018 [cited 2022 Nov 16]. https://www.usaid.gov/documents/1860/usaid-resilience-sahel-enhanced-rise-ii-technical-approach-working-paper.

[39] SOME B (2018). Resilence in the Sahel enhanced gender analysis.

[40] Wegs C, Creanga AA, Galavotti C, Wamalwa E. Community Dialogue to Shift Social Norms and Enable Family Planning: An Evaluation of the Family Planning Results Initiative in Kenya. Bhattacharya S, editor. PLoS ONE. 2016;11(4):e0153907. 10.1371/journal.pone.0153907 PMC4849797 27124177

[41] Shattuck D, Kerner B, Gilles K, Hartmann M, Ng’ombe T, Guest G (2011). Encouraging Contraceptive Uptake by motivating men to communicate about Family Planning: the Malawi Male Motivator Project. Am J Public Health.

[42] Hartmann M, Gilles K, Shattuck D, Kerner B, Guest G (2012). Changes in couples’ communication as a result of a male-involvement Family Planning intervention. J Health Communication.

[43] Challa S, Shakya HB, Carter N, Boyce SC, Brooks MI, Aliou S et al. Associations of spousal communication with contraceptive method use among adolescent wives and their husbands in Niger. Todd CS, editor. PLoS ONE. 2020;15(8):e0237512. 10.1371/journal.pone.0237512 PMC7416918 32776980

[44] Fleming PJ, Shakya H, Farron M, Brooks MI, Lauro G, Levtov RG (2020). Knowledge, attitudes, and practices related to family planning and gender equity among husbands of adolescent girls in Niger. Glob Public Health.

[45] Begum K, Ouédraogo CT, Wessells KR, Young RR, Faye MT, Wuehler SE (2018). Prevalence of and factors associated with antenatal care seeking and adherence to recommended iron-folic acid supplementation among pregnant women in Zinder, Niger. Matern Child Nutr.

[46] Kraft JM, Wilkins KG, Morales GJ, Widyono M, Middlestadt SE (2014). An Evidence Review of Gender-Integrated Interventions in Reproductive and maternal-child health. J Health Communication.

